# Some Additional Experiments upon the Communicability of Tuberculosis in Dogs by Means of Inhalation of Phthisical Sputa

**Published:** 1881-04

**Authors:** P. W. Van Peyma

**Affiliations:** Tueran


### Translations.

### SOME ADDITIONAL EXPERIMENTS UPON THE COMMUNICABILITY OF TUBERCULOSIS IN DOGS BY MEANS OF INHALATION OF PHTHISICAL SPUTA.—BY DR. TAPPEINER, AT TUERAN.

FROM THE GERMAN BY P. W. VAN PEYMA, M. D.

By the first of March I had again collected six healthy dogs, my object being the production of tuberculosis by means of the inhalation of phthisical sputa. I wished also to institute some therapeutical observations upon feeding with the benzoate of soda and with carbolic acid. The dogs were numbered respectively Nos. 0, 1, 2, 3, 4 and 5.

Of these dogs Nos. 1 and 2 were appointed to be fed with benzoate of soda; No. 3 was designated to receive carbolic acid; while Nos. 0, 4 and 5 were selected for comparison and were to remain without medicamental feeding. The feeding began on the seventh of March, an entire week previous to the commencement of inhalation. Dogs Nos. 1 and 2 received daily ten grams of the benzoate of soda, dissolved in water and mixed with the food. No. 3 received daily three pills, each containing six centigrams of carbolic acid and administered in a piece of meat.

In consequence of the drugging, the benzoic dogs soon became dull and ill and refused to eat. The daily dose was accordingly diminshed to five grains, and even with this dose the dogs appeared to suffer and to eat less than formerly. The carbolic dog swallowed his pills as well as his usual quantity of food.

This feeding was continued until the death of the dogs.

The inhalations began March 14th. Each of the six dogs received daily six grams of tubercular sputa mixed with water and evaporated in wooden cages or kennels, of the capacity of one cubic meter. The dogs remained in the cages six hours after the inhalations and were then returned to their usual abode—an airy cellar on the ground level. These inhalations were continued for two weeks, until the 28th of March. On the 2d of April, twenty days after the beginning of the inhalations, the benzoic dog No. 2 died. The animal was much emaciated. The autopsy showed the lungs, liver and spleen free from tubercular infiltration. *The period of incubation was not yet completed.* The eruption of miliary tubercles in dogs is discoverable, for the first, after twenty-three days.

On the 8th of April, twenty-six days after the commencement of the inhalations, the benzoic dog No. 1 died. The subsequent autopsy showed a distinct although not very general infiltration of both lungs. The mucous membrane of the stomach and intestines showed marks of a chronic gastro-enteritis. The cause of death in the benzoic dog No. 2 was a recent and severe gastro-enteritis, with abscesses and hæmorrhagic exavasations in the stomach. On the 10th of April the comparison dog No. 5 was killed. The autopsy showed very general infection of both lungs, nearly double that found in the benzoic dog No. 1. This comparative fact, apparently indicating that the benzoate of soda exercised a restraining influence upon the process of infection, I decided that dog No. 0 should likewise be fed upon this drug. But as No. 1 had shown symptoms of gastro-enteritis on five grams daily, the dose was again diminished one-half, two and one-half grams daily.

On the 14th of April, thirty-two days after the commencement of inhalation, the carbolic acid dog No. 3 was killed. The autopsy disclosed a very great infiltration of both lungs. The animal was not emaciated, on the contrary, his weight had increased one pound, beginning at 27 pounds and ending at 28 pounds.

On the 27th of April the third benzoic dog (No. 0) died, he having been fed two and one-half grams daily since April 12th. The autopsy revealed decided infiltration of both lungs. The animal had lost two pounds in weight, beginning at 11½ and ending at 9. The remaining comparison dog No. 4 became during May somewhat ill and emaciated, but following June 1st he had gained perceptibly, so that when on July 6th he was brought before me, he appeared as lively and well as it is possible for a dog to be. He had clearly already overcome the artificially induced disease. I shall keep him until September, and then see if objectively at the autopsy all traces of tubercle have disappeared.

The results of these experimental investigations have positively corroborated the fact of the induction of tuberculosis in dogs by means of simple inhalations of evaporating phthisical sputa. This I had already established in Munich, and later in Meran and Berlin, so that up to the present time not a single experiment has given negative results. But the hope that the benzoate of soda or carbolic acid had a positive effect, either prophylactic or healing, is unhappily not confirmed, but on the contrary has been disproved. My dogs have for an entire week, previous to the inhalation of the tubercular sputa, been fed upon sufficiently large doses of carbolic acid and the benzoate of soda that the sytem was certainly saturated. And yet the infiltration occurs in all the dogs. The drugs when administered were continued to the time of death, notwithstanding which the autopsy proves infiltration as general as that found in unmedicated dogs.—*Berliner Klinische Wochenschrift, 1880.*

